# Nonlinear dynamics based digital logic and circuits

**DOI:** 10.3389/fncom.2015.00049

**Published:** 2015-05-15

**Authors:** Behnam Kia, John. F. Lindner, William L. Ditto

**Affiliations:** ^1^Applied Chaos Lab, Department of Physics and Astronomy, University of Hawai'i at MānoaHonolulu, HI, USA; ^2^Physics Department, The College of WoosterWooster, OH, USA

**Keywords:** Boolean logic, nonlinear dynamics, dynamics based computing, noise robustness, dynamical coupling, chaos computing, ternary logic gate, multiple-valued logic circuits

## Abstract

We discuss the role and importance of dynamics in the brain and biological neural networks and argue that dynamics is one of the main missing elements in conventional Boolean logic and circuits. We summarize a simple dynamics based computing method, and categorize different techniques that we have introduced to realize logic, functionality, and programmability. We discuss the role and importance of coupled dynamics in networks of biological excitable cells, and then review our simple coupled dynamics based method for computing. In this paper, for the first time, we show how dynamics can be used and programmed to implement computation in any given base, including but not limited to base two.

## Introduction

**Figure 1:**
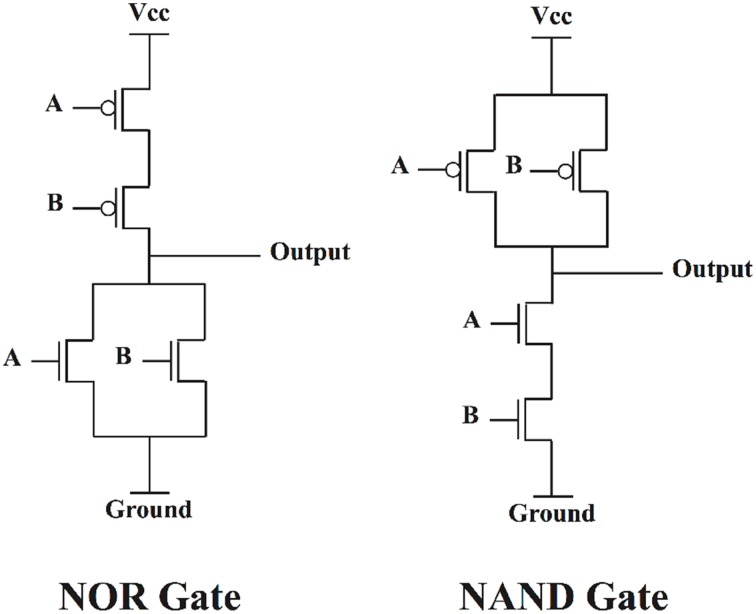
**Different arrangements of transistors, acting as switches, implementing different functions**.

There are fundamental differences between how biological neural networks and human-made computer systems perform computation. Modern computer systems are based on Boolean logic and Boolean circuits. A Boolean circuit is an arrangement of bistable switches, where the switches are turned on or off based on the incoming data or control inputs. As an example, in Figure 1 two different arrangements of transistors are depicted which implement two different functions, NOR and NAND operations. In these circuits, the transistors operate as a switch, and turn on or off depending on the incoming data. In a NAND gate, when at least one of the signals is “0,” the corresponding PMOS transistors that are controlled by this “0” signal will switch on, and the output will be connected to Vcc, representing state “1.” Or in a NOR case, when at least one of the signals is “1,” the correspond NMOS transistors will switch on, and the output will be connected to the ground, representing state “0.” The conventional Boolean circuits are nothing more than such circuits of switching transistors.

In these arrangements, there is virtually no dynamics involved, except a simple switching process, and the entire information processing and computing are performed based on the structural connectivity and arrangement of the switches. But in the brain and biological neural network the information processing is not just a product of structural and anatomical connections of neurons, but also dynamical as well (McKenna et al., 1994; Fox et al., 2005; Canolty et al., 2007; Izhikevich, 2007; Sporns, 2011). Each neuron itself is a nonlinear dynamical system that illustrates a broad range of dynamics such as different types of bifurcation (Izhikevich, 2007). Furthermore, different neurons within a network are dynamically coupled together and phenomena such as synchronization (Varela et al., 2001; Izhikevich, 2007), neuronal avalanches (Plenz and Thiagarajan, 2007), and correlation and anticorrelation (Fox et al., 2005) occur among them.

In this paper we follow this argument that dynamics is one the main missing components in conventional logic circuits, and this lack of dynamics cripples conventional computing systems reaching the performance and robustness levels of the brain and biological neural systems. We briefly overview dynamics based computing, and show how dynamics (1) can be utilized to achieve different functions and hopefully even plasticity (2) can be used to achieve robustness against noise, (3) can unshackle us from the hegemony of binary logic.

## Nonlinear dynamics as the source of different behaviors

A neuron, or any other excitable cell, can remain resting or can fire different patterns of action potentials, such as regular spiking, intrinsically bursting, subthreshold oscillations, or chaotic firing (Izhikevich, 2003; Qi et al., 2013). Neurons, or any other excitable cells, are nonlinear dynamical systems, and their broad range of behaviors are attributed to their nonlinearity. Many such neural phenomena, such as a neuron switching between rest mode and regular spiking mode, can be modeled and explained in terms of dynamical systems theory, such as bifurcation phenomena (Izhikevich, 2007; Qi et al., 2013).

In conventional Boolean circuits, the systems are stripped from their natural dynamics, and are controlled and reduced to act as simple on/off switching circuits, as shown and explained in Figure 1.

**Figure 2:**
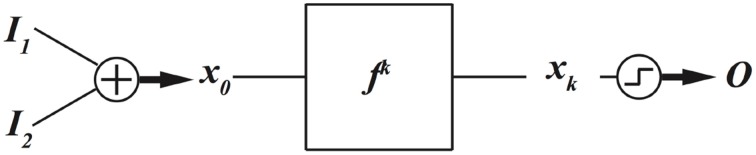
**A schematic model for dynamics based computing**.

In dynamics based computing, we bring back dynamics to computing (Sinha and Ditto, 1998, 1999; Munakata et al., 2002; Sinha et al., 2002a,b; Pourshaghaghi et al., 2009; Crutchfield et al., 2010; Kia et al., 2011a,b, 2014a). Schematic diagram of a dynamics based computing model is depicted in Figure 2.

In this model, the aim is to implement a two-input, one-output, combinational digital function, such as AND, OR, or XOR gate. Two data input, *I*_1_ and *I*_2_, are added and mapped to an initial condition of a dynamical system *f*. The dynamical system evolves *k* times, and the output 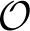 is decoded from the final state *x*_*k*_ using a threshold mechanism. Notice that mathematically speaking, a function is a mechanism that maps the inputs to the outputs. A dynamical system maps its current state to future states. As a result, the dynamical system can be considered as a realization of a function. Now the task is reduced to finding which functions a given dynamical system can implement, and much more importantly, how a given dynamical system can be dynamically programed to implement different functions. This has been the focus of many of our and others research works on chaotic and dynamics based computing (Sinha and Ditto, 1998; Munakata et al., 2002; Peng et al., 2008, 2011; Murali et al., 2009; Campos-Cantón et al., 2010; Kia et al., 2011a,b; Li et al., 2013).

In this paper, we review three of main categories for chaos computing, and illustrate them using simple models and pictures. Each of these techniques introduces a systematic method to reprogram a dynamics-based system to implement many different types of computation. This provides the chaos-based computing system with *flexibility* and *variability* and this opens the door to plasticity, where each chaos-based system can adapt through dynamical reconfiguration to different conditions. In this paper, we do not specifically present an autonomous adaptation method for these chaos-based systems; but the potential is present for both plasticity and adaptation. We and others are working to incorporate computational intelligence mechanisms into these chaos-based systems to create adaptable chaos-based computing systems.

### Programing with bias values

**Figure 3:**
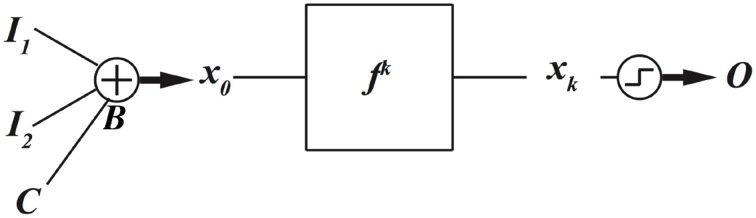
**Control value *C* biases initial conditions of a nonlinear dynamical system to program the dynamical system to implement different digital functions**.

The initial condition of the dynamical system, which is produced from the data input, can be biased differently as a technique to implement different functions. Nonlinear, chaotic dynamical systems are sensitive to initial conditions; as a result these bias values change the future evolution of the chaotic system, and therefore new functions can be implemented. The block diagram for this method is depicted in Figure 3. As a numerical example, assume the nonlinear dynamical equation is famous logistic map

(1) $$x_{n+1}=f_{\lambda }\left[x_{n}\right]=\lambda x_{n}(1-x_{n}),$$

where λ is a parameter, and *x*_*j*_ ∈ [0, 1]. In this example we set λ = 4, which puts the dynamical system of Equation (1) in a chaotic regime.

Assume the incoming data inputs and control input are encoded as the initial condition of the logistic map as

(2) $$x_{0}=ℰ\left[I_{1},I_{2}\right]=\frac{I_{1}+2I_{2}}{4}+B,$$

where *B* is the control value that biases the initial condition. Notice that the control value should not be too large to bias the initial condition outside of the [0, 1] interval of the dynamical system of Equation (1). The encoding map of Equation (2) can be interpreted as a simple digital to analog convertor; however, for encoding data inputs to an initial condition we do not need an exact digital to analog convertor. Any function that maps digital inputs to an analog value can be used as an encoding map, but it is more efficient if the mapped initial conditions are equally spaced.

The output 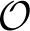 can be decoded from the final state of the dynamical system as

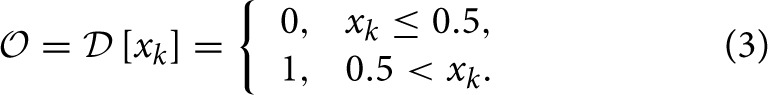



When the nonlinear dynamical system is chaotic, noise can be problematic. In such cases, the evolution time of the nonlinear system should be adjusted so that it is long enough for bias values to change the future state of the chaotic system, but not too long so that even small noise can change the future states as well.

**Figure 4:**
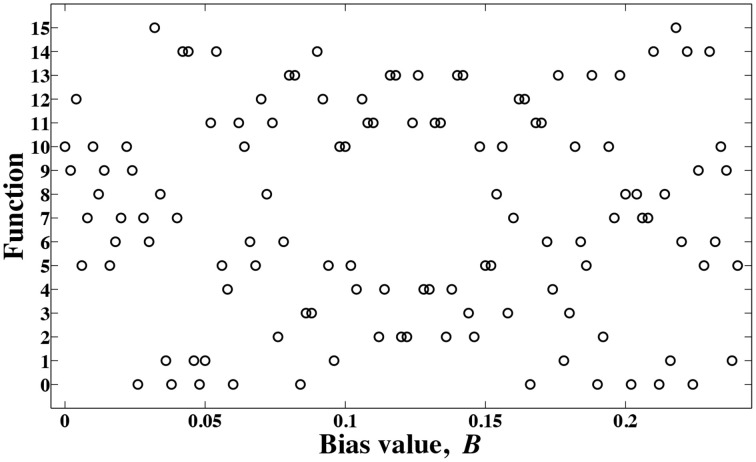
**Different functions are obtained for different bias values B**.

The resulting functions for different bias values are presented in Figure 4. To produce these results, we have assumed the evolution time *k* = 8, and varied the bias value with an incremental step size of Δ*B* = 0.002. Some intermediate bias values may result in different functions. Notice that there are 16 different two-input, one-output digital functions, and not all of them have well-known names such as AND, XOR gates. As a result, here we use a simple labeling technique to name these different functions. When different combinations of data inputs, (0,0), (0,1), (1,0), and (1,1) are fed to the computing model of Figure 3 and the outputs are 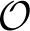
_0_, 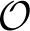
_1_, 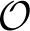
_2_, and 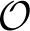
_3_ respectively, where 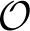
_*i*_ ∈ {0, 1}, then this function is called function number 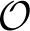
_0_ × 2^0^ + 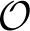
_1_ × 2^1^ + 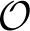
_2_ × 2^2^ + 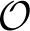
_3_ × 2^3^. As a result, each function will have a unique name between 0 and 15. For example, a function with all 0 output would be function number 0, and a function with all 1 output will be function number 15.

### Programing with parameters

**Figure 5:**
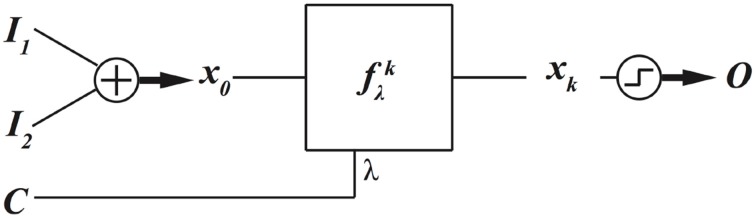
**Parameters of a nonlinear dynamical system can be adjusted to program the dynamical system to implement different digital functions**.

Assume *f*_λ_ is a nonlinear dynamical equation, and λ is a bifurcation parameter. The behavior of a nonlinear dynamical system qualitatively and quantitatively changes with the change of bifurcation parameter. This feature can be utilized to program a dynamical system to implement different digital functions. The block diagram of this method is depicted in Figure 5.

In this block diagram, there is an additional input, the control value *C* = λ, to set the parameter of the dynamical system. Different parameters can lead to the realization of different functions. As a numerical example, assume the same nonlinear dynamical equation of Equation (1). Assume the data inputs are encoded as the initial condition of the logistic map by

(4) $$x_{0}=ℰ\left[I_{1},I_{2}\right]=\frac{I_{1}+2I_{2}}{4}+0.123,$$

and we use the same decoding map of Equation (3) to produce the outputs.

**Figure 6:**
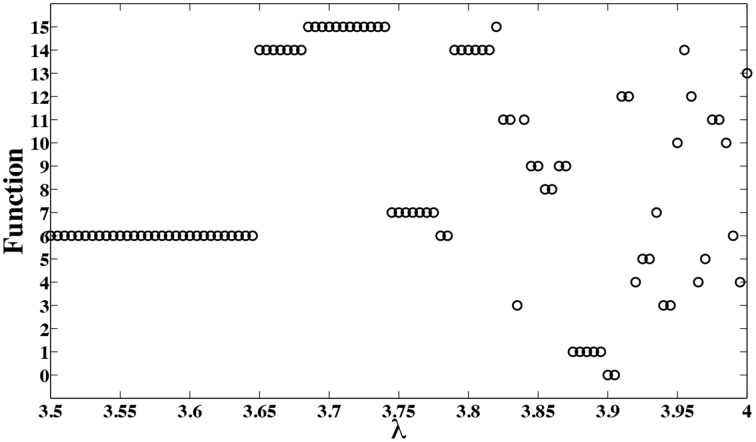
**Different functions are obtained for different bifurcation values λ**.

**Figure 7:**
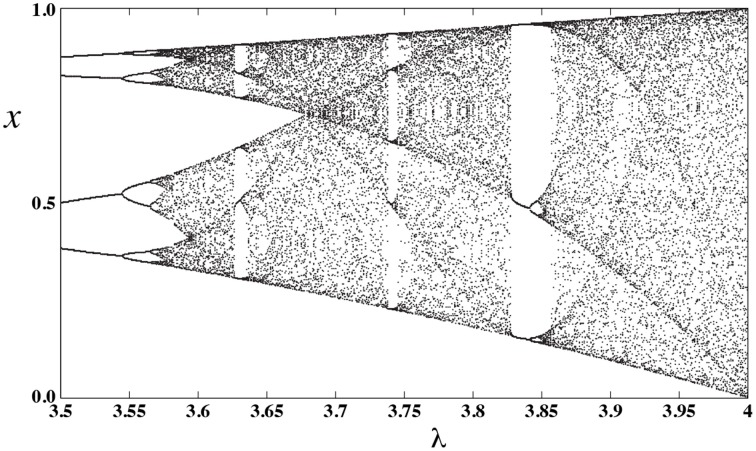
**Bifurcation diagram of logistic map**.

Now, by changing the bifurcation parameter λ, the computing model of Figure 5 can be programmed to implement different functions as is shown in Figure 6. To produce these results, we have assumed *k* = 8, and varied the bifurcation parameter λ with an incremental step size of Δλ = 0.002. Notice that other intermediate values of λ can result in different functions. There is a rough correlation between bifurcation diagram of logistic map, Figure 7, and the functions that can be implemented at different bifurcation values, Figure 6. In Figure 7, lower λ values, the dynamics of logistic map is quite simple and there is just a stable periodic orbit. As λ increases, a period doubling bifurcation starts and eventually it leads to the full chaotic regime. In Figure 6 we observe that for lower values of λ, where there are long bifurcation intervals, identical functions are implemented for different values of parameter λ. But as λ increases and period doubling bifurcation occurs at shorter intervals, nearby λ values result in different functions. And the extreme case is when we enter into the chaotic regime, where even a slight change of the bifurcation parameter can change the implemented function.

In Section Dynamical coupling and synchronization for robust dynamics based logic we will discuss the evolution time *k* and the noise effects.

### Programing with evolution time

**Figure 8:**
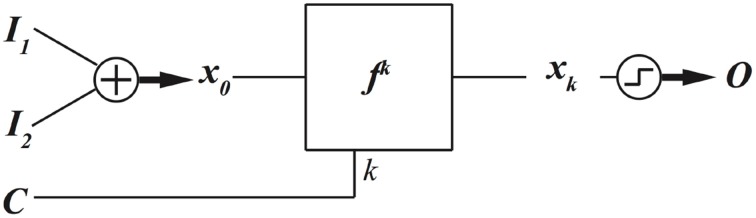
**Evolution time of a chaotic dynamical system can be adjusted to program the chaotic dynamical system to implement different digital functions**.

When a nonlinear dynamical system is in a chaotic regime, it never repeats the same patterns. This means that a chaotic dynamical system can produce different outputs at different evolution times. This can be an additional method to program a chaotic dynamical system to produce different functions. The block diagram for this method is depicted in Figure 8.

**Figure 9:**
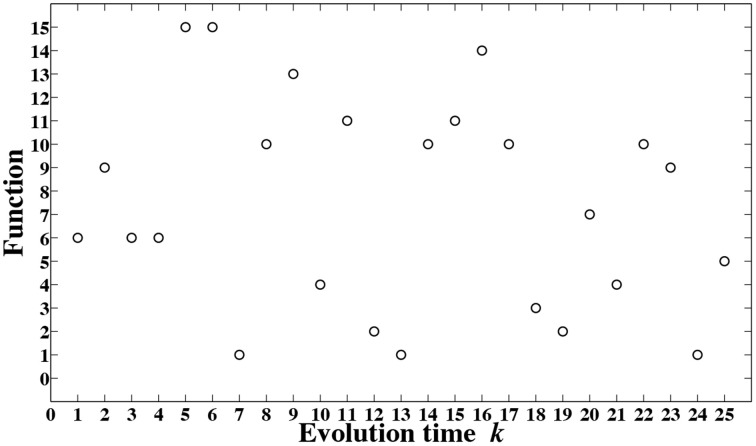
**Different functions are obtained for different evolution time *k***.

The resulting functions for different evolution time, control value *C* = *k*, are depicted in Figure 9. To produce these results, we have used the dynamical system of Equation (1), set λ = 4 to put the logistic map in a chaotic regime and used the encoding and decoding maps of Equation (4) and Equation (3), respectively.

In Sections Programing with bias values, Programing with parameters, and Programing with evolution time, we summarized three different methods to program a dynamical system to implement different functions, one or any combination of these three methods can implement and program dynamics based computing.

## Dynamical coupling and synchronization for robust dynamics based logic

Gap-junctional coupling can synchronize electrically active cells, such as brain neurons, heart pacemaker cells, or pancreatic β-cells (Sherman and Rinzel, 1991). Different roles have been suggested for synchronization within biological networks. Neural binding (Engel and Singer, 2001) and selective attention (Womelsdorf and Fries, 2007) are examples of hypothesized roles for synchronization in neural networks.

It is also hypothesized that synchronization of biological cells, realized by active, dynamical coupling among cells, reduces the effects of noise and unwanted fluctuations. For example, it was shown that cells in an islet of Langerhans, which are electrically coupled by gap junctions, burst synchronously (Meissner, 1976; Eddlestone et al., 1984; Sherman and Rinzel, 1991; Loppini et al., 2014), whereas isolated cells exhibit disorganized spiking.

Similarly, in the context of neural networks, it was suggested that collective enhancement of precision, or simply noise reduction, is another role for synchronization (Tabareau et al., 2010; Bouvrie and Slotine, 2011; Medvedev and Zhuravytska, 2012). For example, it is hypothesized that synchronization may help protect interconnected neurons from the influence of intrinsic neuronal noise (Tabareau et al., 2010).

**Figure 10:**
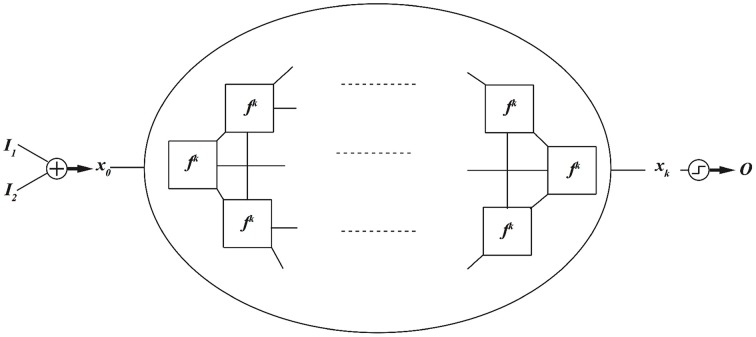
**Coupled dynamical systems, implementing robust to noise computation**.

We have shown that our dynamical logic circuits can also be coupled together to implement a robust to noise computation. The block diagram of this method is depicted in Figure 10.

**Figure 11:**
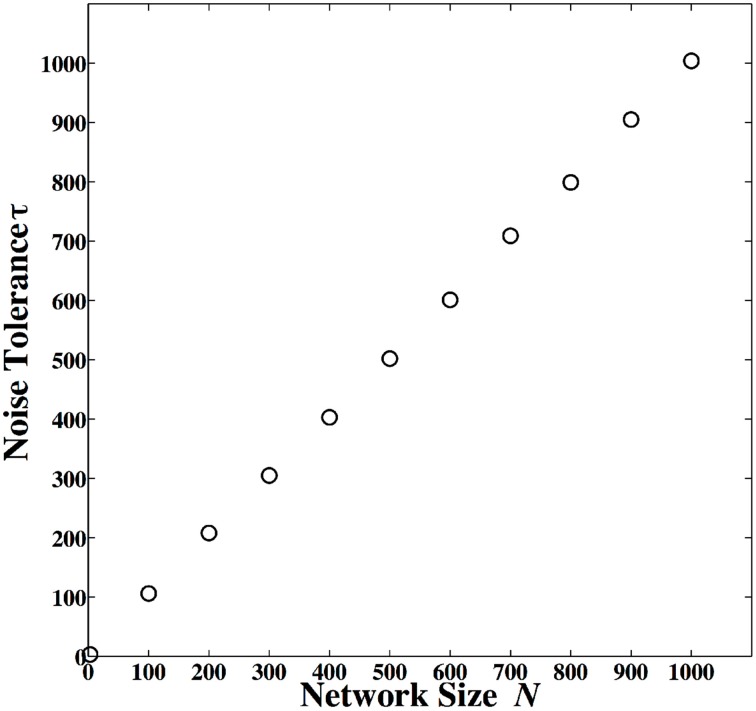
**Noise tolerance for coupled dynamics based computing for different network sizes**.

In this block diagram, rather than a single dynamical system, a series of dynamically coupled identical dynamical systems is utilized for computation. All dynamical systems in this coupled lattice receive the same input data, which initializes all dynamical systems to the same initial condition. The final state of one of the dynamical systems in the lattice is used to decode the output. This coupled lattice of dynamical system can mitigate local noise. Local noise is a noise that is statistically independent from one spatial location (here dynamical system) to another. We have shown that under coupled dynamics, noise from different nodes diffuses through the lattice and attenuate the effects of noise in other nodes (Kia et al., 2014b, 2015). This is roughly similar to hypostasis in the context of excitable cells where the coupled dynamics enhances the precision and reduces the noise. We have tried different coupling mechanisms, and we have obtained similar results. More specifically, we have shown that in a coupled map lattice of size *N*, when all nodes are globally and optimally coupled according to Kaneko's coupled map lattice model, the noise content in the lattice reduces by a factor of *N*. We have utilized this feature to implement robust-to-noise computing based on coupled dynamics (Kia et al., 2014b), where we simulated small sized networks. But we know that in the brain neurons can be connected and coupled to thousands of other neurons. In this paper, we repeat the same simulation, but for larger network sizes. We define noise tolerance as

(5) $$\sigma =\sigma_{C}^{2}/\sigma_{S}^{2},$$

where σ^2^_*S*_ is the maximum variance of additive noise that a single-map based chaos computing system can tolerate without exceeding a specified error rate, and σ^2^_*C*_ is the maximum variance of additive noise that a coupled dynamics-based chaos computing system can tolerate without exceeding the same specified error rate. We use a Monte Carlo simulation to estimate the noise tolerance for different lattice sizes and the results are presented in Figure 11. We observe that when 1000 dynamical systems are coupled together, the resulting coupled dynamics-based computing will be 1000 times more robust to noise. And this is correct for different values of network size. The encoding and decoding is exactly the same as Equation (2) and Equation (3). But the difference is that now rather than having a single map of Equation (1), we have *N* maps of Equation (1), globally coupled together as

(6) $$x_{i+1}^{j}=(1-\varepsilon )f(x_{i}^{j})+\frac{\varepsilon }{N-1}\sum_{p\neq j}f(x_{i}^{p})+\sigma_{C}\delta_{i+1}^{j},$$

where *x*^*j*^_*i*_ is the dynamical state of the *j*th node in the network at time *i*, ε is the coupling parameter, σ^2^_*C*_ is noise variance, and δ^*j*^_*i* + 1_ ≈ *N*(0, 1) is normal Gaussian local noise with zero mean and unit variance. We have shown analytically and in simulation that ϵ = (*N* − 1)/*N* is the optimal parameter value, which results in maximum noise tolerance. More specifically, we have calculated noise tolerance for different parameter values, and observed that ϵ = (*N* − 1)/*N* produces the maximum noise tolerance. Also, analytically we have calculated the variance of evolved noise over evolution time, and shown that ϵ = (*N* − 1)/*N* minimizes the variance of evolved noise. For decoding output, we can choose any node from the network and decode the output based on its final state. For further details about coupled dynamics based computing, optimal parameter values for noise mitigation, and the methods we have used to obtain the results of Figure 11, please refer to our earlier work (Kia et al., 2014a).

## Computation in arbitrary base β

Conventional digital circuits are implemented with bistable switches that have two states: on or off. As a result, such circuits are suitable for binary computations, where each signal has two states, “0” or “1.”

There have been different efforts to implement digital circuits in bases other than binary. As an example, multi-threshold carbon nanotube field effect transistors (CNTFETs) were utilized to design a ternary logic gate (Lin et al., 2011; Moaiyeri et al., 2013). Or three-state quantum dot gate field effect transistors (QDGFETs) were used to design ternary logic combinational circuits (Karmakar et al., 2013).

When we utilize dynamical systems to implement digital computation, we are not restricted to on/off switches and their binary states. Therefore, in principal we can implement digital functions in any base. In dynamics based computing, the final output is the symbol that is assigned to the final state of the dynamical system. One can always use a multi-symbol partitioning and perform dynamics based computation at any desired base. However, it has to be noted that even though symbolizing a dynamical system is arbitrary, the selection of too many partitions, or using an inefficient partitioning, can reduce the efficiency of dynamics based computing. The main purpose of utilizing nonlinear dynamics for computation is to harness the rich intrinsic patterns within the nonlinear dynamics. Symbolizing a dynamical system can reduce the number of such intrinsic patterns, if an inefficient partitioning is used.

**Figure 12:**
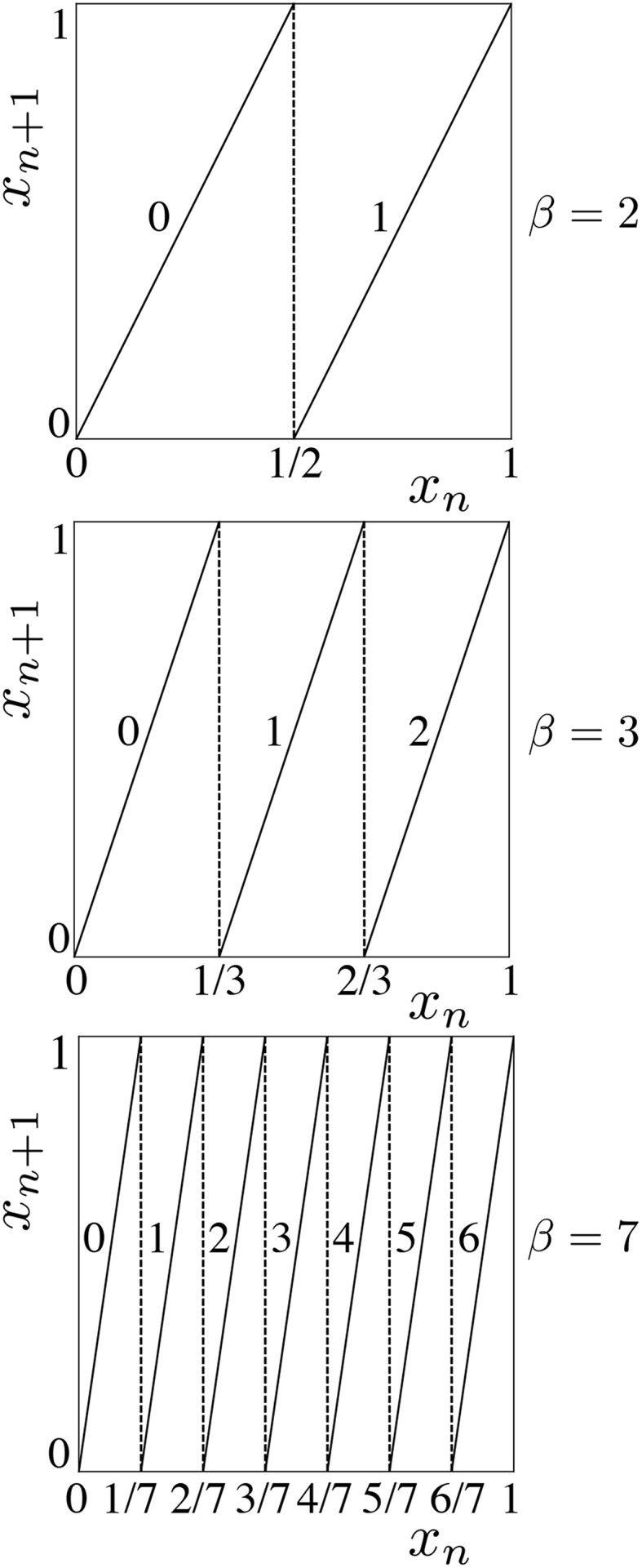
**The 1-*D* map of Equation (7) for different β values**.

As an example, consider the chaotic 1-*D* sawtooth map, also known as shift map,

(7) $$x_{n+1}=\beta x_{n}(mod1)$$

on unit interval [0, 1], where β is a parameter, as shown in Figure 12 (top) for β = 2. Selection of threshold 0.99 to partition the state space into (0, 0.99) for symbol 0 and (0.99, 1) for symbol 1 does not preserve the entropy of the original map. A symbolic representation of an orbit of the sawtooth map with this partition will result in many consecutive 0s sparsely separated by 1s. If an external observer who is not aware of the exact initial condition of this chaotic system watches the symbolic time series, he can predict that the next symbol would be 0, and in many cases he will be correct. This partitioning reduces the unpredictability, which is also known as information from Shannon's communication point of view. Entropy is a classic measure to quantify the amount of information in a system. A partition is called a generating partition if it preserves the Kolmogorov-Sinai entropy of the dynamical system (Collet and Eckmann, 2007) after symbolizing the orbits. For 1-*D* maps, separating the intervals at the critical points of the map creates optimal generating partitions (Bollt, 2003; Collet and Eckmann, 2007).

By changing the sawtooth slope parameter β, we can adjust the cardinality of the generating partitions. If β is an integer number, then cardinality of the generating partition will be β as well. The 1-*D* map of Equation (7) is plotted for β = 2, 3, and 7 in Figure 12(top), (middle), and (bottom), respectively. This enables a parametric dynamical system to be programmed to naturally operate at different bases.

As an example, we show here how by selection of β = 3 we can perform ternary—base three—computation. First consider implementing ternary negation. The truth table for ternary negation is shown in Table 1. *I* is the single input to the ternary negation gate, and 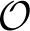 is the output, and there are three symbols, 0, 1, and 2.

**Table 1:** **Ternary Negation**.

*I*	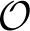
0	2
1	1
2	0

**Figure 13:**
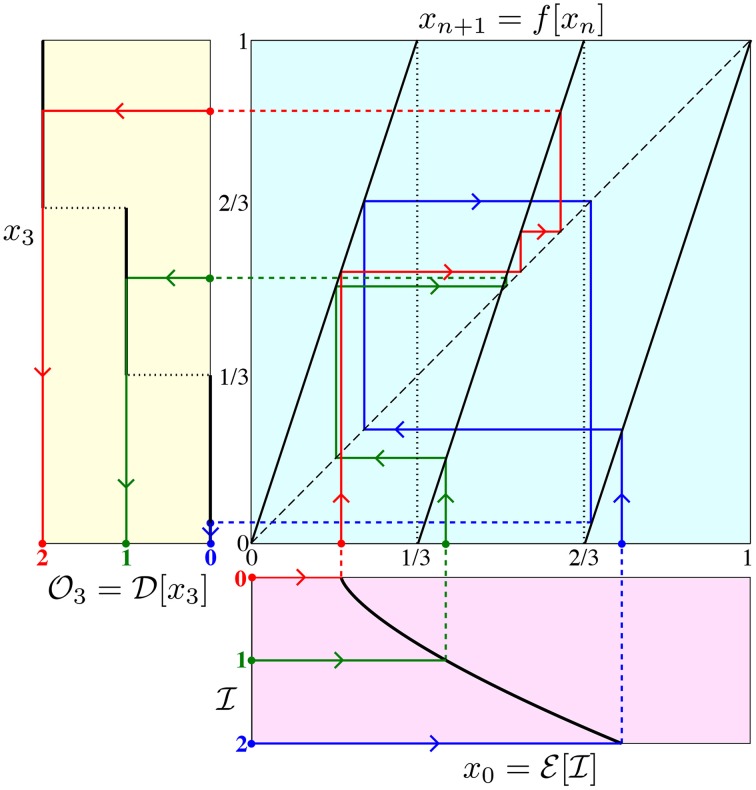
**Dynamics-based realization of a ternary negation gate.** Arrowheads lead from encoding the input (bottom magenta graph) to iterating the nonlinear map (top right cyan graph) to decoding the output (left yellow graph).

The encoding map we use here is

(8) $$x_{0}=ℰ\left[I\right]={(\frac{I}{3})}^{1.42}+0.18$$

and the decoding map to produce the output is 
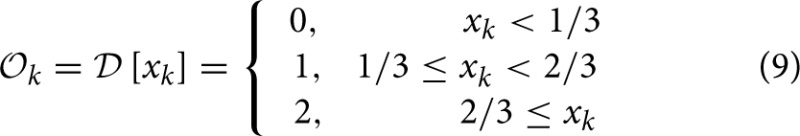
 where *k* = 3. The base-three encoding map of Equation (9) is very similar to the base-two encoding map of Equation (2), where *I*/3 represents a simple digital to analog convertor, and 0.18 is the bias value to program the dynamical system of Equation (7) to implement a ternary negation gate of Table 1. But it differs from the Equation (2) encoding map because of its nonlinearity. The reason is an artifact of the strong symmetry between a linear base-three encoding method and the dynamics of the Equation (7) sawtooth map. This is a very special case, and it normally does not happen in dynamics based computing, but in this specific dynamical equation, the encoding map is basically the reverse of the chaotic map, and therefore they cancel out each other's operation. But a slight nonlinearity in encoding map, resolves the issue. Figure 13 shows a dynamics-based, base-three negation operation, which maps “0” to “2,” “1” to “1,” and “2” to “0.”

In the next example, we show how a two-input, one-output ternary AND operator, also known as minimum operator, can be dynamically implemented. The encoding map we use here is

(10) $$x_{0}=ℰ\left[I_{1},I_{2}\right]={(\frac{I_{1}+3I_{2}}{9})}^{1.42}+0.291$$

that maps two data inputs *I*_1_ and *I*_2_ to an initial condition and decodes the output using the Equation (9) decoding map. The results are listed in Table 2, where 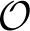 is the output of computation.

**Table 2:** **Ternary AND**.

*I*_1_	*I*_2_	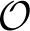
0	0	0
0	1	0
0	2	0
1	0	0
1	1	1
1	2	1
2	0	0
2	1	1
2	2	2

By selecting any other integer values for parameter β, the dynamical system of Equation (7) “*naturally”* and *“faithfully”* performs computation in base β.

## Conclusions

Compared to the brain and biological neural networks, dynamics is one of the main missing elements in Boolean circuits. Dynamics plays a crucial role in the brain, whereas in conventional Boolean circuits the dynamics is virtually nonexistent (except as a simple switching process). In this paper we reviewed our dynamics based computing, and showed how dynamics can be utilized to implement logic circuits. Noise robustness in neurons and other excitable cells is partially attributed to synchronization and coupling between different neurons. In parallel to these biological observations, we showed how dynamics based computing systems can be similarly coupled to enhance and improve their noise robustness. Finally, for the first time, we showed how nonlinear systems can be programmed to naturally implement computation at different bases. We used a parametric dynamical system, where changing the parameter qualitatively changes the mapping between inputs and outputs, and a different partition with a different cardinality fits the dynamics better. As a result, changing such parameters enable dynamical systems to naturally perform computation at different bases.

The focus of this paper was mostly on abstract models and ideas for dynamics based computing, and no exact physical implementation was introduced. However, it has to be noted that we do not need to introduce or add or construct nonlinear dynamics, rather the nonlinear dynamics is naturally there, and any transistor, or transistor circuit is governed with nonlinear dynamical equations. We even argue that it is the conventional Boolean circuits that are unnatural and abstract, where the intrinsic nonlinear dynamics of transistors are being controlled and suppressed, and they are reduced to simple on/off switches.

We have introduced different proof of concept circuit implementations for dynamics based computing, and these ideas have been verified experimentally (Murali et al., 2003, 2005; Pourshaghaghi et al., 2009, 2010). We have recently fabricated an integrated circuit for dynamics based computing. In this integrated circuit, the intrinsic nonlinearity of transistors and their rich dynamics are utilized to implement different functions. As a result, the same circuit, which is constructed with very few transistors, is dynamically programmable to implement different functions. This fabricated integrated circuit is under testing and measurement now, and the results will be published in a future research paper.

### Conflict of interest statement

The authors declare that the research was conducted in the absence of any commercial or financial relationships that could be construed as a potential conflict of interest.
